# Photoinduced post-modification of graphitic carbon nitride-embedded hydrogels: synthesis of 'hydrophobic hydrogels' and pore substructuring

**DOI:** 10.3762/bjoc.17.92

**Published:** 2021-05-21

**Authors:** Cansu Esen, Baris Kumru

**Affiliations:** 1 Max Planck Institute of Colloids and Interfaces, Department of Colloid Chemistry, Am Mühlenberg 1, 14476 Potsdam, Germany

**Keywords:** hydrophobic hydrogel, photoactive hydrogels, photomodification, pore modification, soft materials

## Abstract

Hydrogels are a special class of crosslinked hydrophilic polymers with a high water content through their porous structures. Post-modifications of hydrogels propose an attractive platform so that a variety of fresh functions, which are not arising from initial monomers, could be accessible on a parental network. Photoinduced post-modification of hydrogels by embedding semiconductor nanosheets would be of high interest and novelty. Here, a metal-free semiconductor graphitic carbon nitride (g-CN)-embedded hydrogel as an initial network was synthesized via redox-couple initiation under dark conditions. Post-photomodification of so-formed hydrogel, thanks to the photoactivity of the embedded g-CN nanosheets, was exemplified in two scenarios. The synthesis of ‘hydrophobic hydrogel’ is reported and its application in delayed cation delivery was investigated. Furthermore, pores of the initial hydrogel were modified by the formation of a secondary polymer network. Such a facile and straightforward synthetic protocol to manufacture functional soft materials will be of high interest in near future by the means of catalysis and agricultural delivery.

## Introduction

Popularity of hydrogels arises from their structural similarity to natural tissues, meaning that they are stable networks with high water content [1–3]. The simplest synthesis of hydrogels can be conducted in an aqueous solution of a water-soluble monomer and crosslinker (bi- or more functional) in the presence of an initiator (generally radical initiation). Since then, many synthetic routes have been developed in order to synthesize artificial matter that mimics the performance of natural tissues. Therefore, many reinforcement methods have been suggested, i.e., host–guest interactions [4–5], double network formation [6–8], and reinforcer addition [9–12]. The potential of hydrogels is beyond biomaterials, currently these aqueous soft materials are prime candidates in agricultural delivery systems as well [13–14].

In the era of sustainability, utilization of sunlight is of great importance [15]. Metal-containing and mostly toxic and non-sustainable semiconductors are slowly being replaced by a new generation of semiconductors. Graphitic carbon nitride (g-CN) is a metal-free polymeric semiconductor that is mainly composed of carbon and nitrogen elements by tri-*s*-triazine, triazine imide, or heptazine repeating units [16–18]. g-CN represents a family of materials where variety of synthetic routes can be applied to form photoactive matter with altered properties, i.e., monomer supramolecular assembly to attain a monomer complex prior to carbonization results in enhanced porosity and photoactivity since ordered structures are formed, and a detailed overview has been reported by Shalom et.al. [19]. Facile tunability has rendered g-CN to be applied in visible-light-induced catalytic reactions such as water splitting [20–22], pollutant degradation [23–26], CO_2_ reduction [27–29], photonics [30–31] and polymer synthesis [32–35].

Integration of g-CN into hydrogels has been popularized in the last four years, where g-CN nanosheets can be implemented into hydrogels through embedding [36] or covalent binding [37–38] for the target application such as reinforced hydrogels [39] and hydrogels for photoredox-based applications [40–41].

**Scheme 1:**
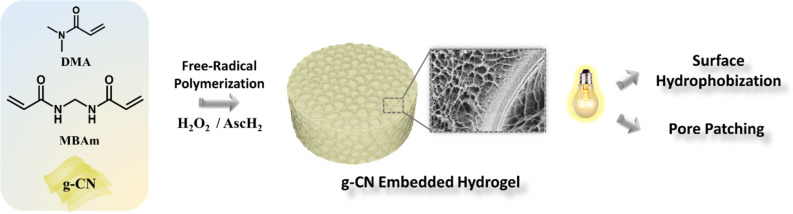
Schematic overview of g-CN-embedded hydrogel fabrication and its subsequent photoinduced post-modifications.

Hydrogel post-modification via semiconductors induced by visible light would be an appealing haven. Herein, we demonstrate the photoactive g-CN nanosheet addition to hydrogels through embedding, which will be the anchoring point that grants an access to photoinduced post-modification methods. The effectiveness of this strategy will be demonstrated via a photoinduced transformation of a hydrophilic skeleton to a hydrophobic network. Furthermore, freeze-dried hydrogel will be subjected to a subsequent photoinduced pore patching (Scheme 1).

## Results and Discussion

Under the scope of this study, which is divided into two parts, the hydrogel denoted as HGCM was utilized as the main substrate for both sections. To prepare HGCM, firstly graphitic carbon nitride was synthesized from the thermal treatment of a cyanuric acid–melamine supramolecular complex (CM) [42]. The resulting yellow powder was ultrasonicated in water to obtain a g-CN aqueous colloidal dispersion. The freshly prepared CM/water colloidal dispersion was mixed with water-soluble monomer (*N*,*N*-dimethylacrylamide, DMA) and crosslinker (*N*,*N’*-methylenebisacrylamide, MBA) followed by the addition of the redox couple, ascorbic acid/hydrogen peroxide, respectively. The mixture was immediately placed in a Petri dish to complete the gelation via free radical polymerization under dark conditions. After 3 hours, the resulting hydrogel was purified with water to remove the unreacted species (monomers and redox mediators), then it was freeze-dried overnight (HGCM). Despite the fact that g-CN-based hydrogels are appealing as g-CN nanosheets can be employed as photoinitiators to form covalent species, in this study external initiators were employed to embed g-CN nanosheets within hydrogel network [36]. In addition, a comparative sample was prepared by the same procedure in the absence of g-CN.

### Hydrophobic hydrogels

**Scheme 2:**
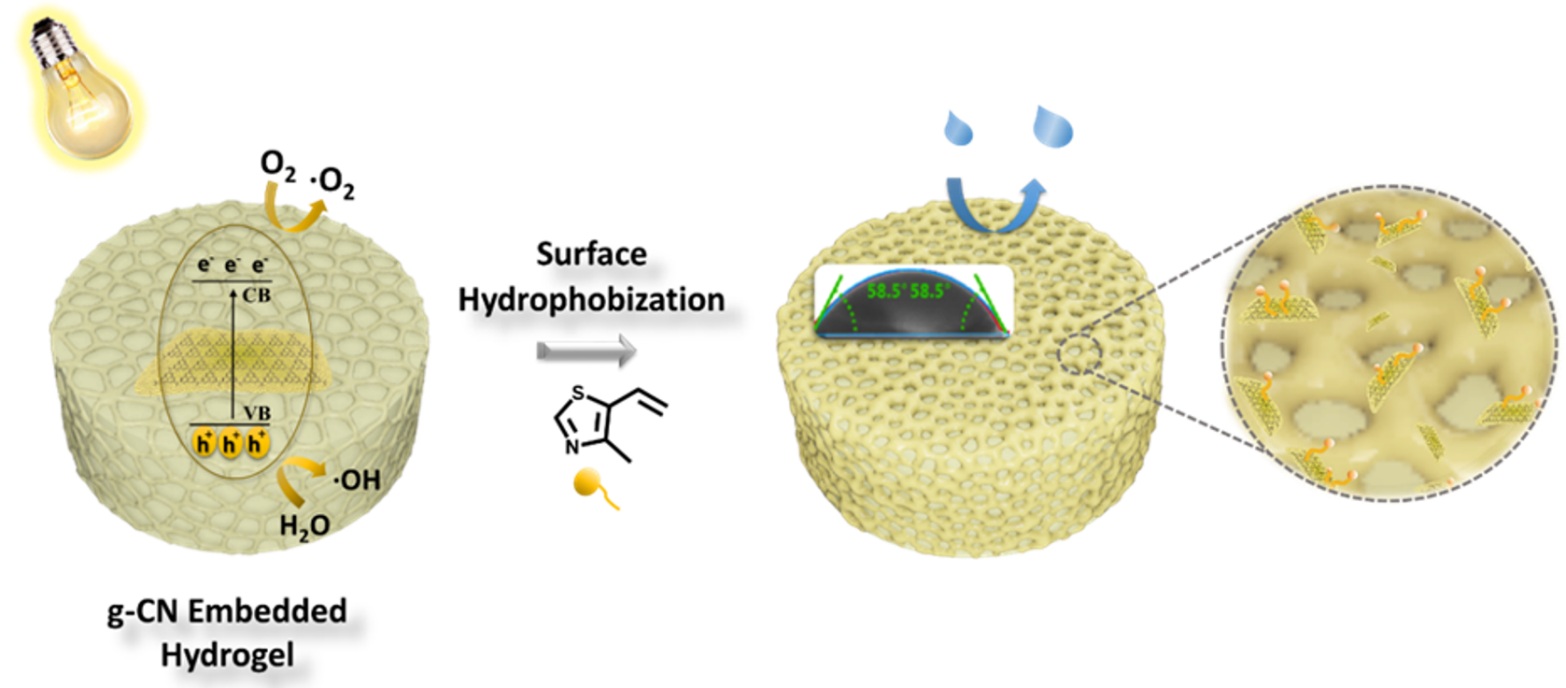
Hydrophobic hydrogel via photoinduced surface modification over embedded g-CN nanosheets in hydrogel network.

Recently, utilization of hydrogels in nutrient delivery in agricultural science and long-term drug delivery exhibits a significant interest [43]. Most of these methods require aqueous formulations to be delivered over a certain amount of time, yet a hydrophobicity for a long-term open-air application must be possessed in order to prevent the drying and a loss of a part [44]. Hydrophobic hydrogel is a novel concept that administers the surface properties of the initial network. In this section, we will propose a straightforward photo-based surface modification to introduce hydrophobicity on a hydrophilic network by taking advantage of the photoactive g-CN nanosheets. As explained in the preparation section, the resulting HGCM was immersed in the hydrophobic monomer 4-methyl-5-vinylthiazole, denoted as vTA, then exposed to visible light irradiation to initiate an in situ surface photomodification. Extensive studies over the last years demonstrated photoinduced g-CN surface modification methods through a photoredox system. vTA, which is a common food additive to donate a nutty taste, has previously shown a significant hydrophobization effect on bulk g-CN [45], so that a similar strategy is targeted for the present case in hydrogel systems (Scheme 2). After adequate light irradiation followed by a facile purification step, the resulting sample (HGCM-vTA) and HGCM were investigated via solid-state analysis to evaluate the vTA incorporation, and microscopy techniques were employed to examine the impact on their morphology. In terms of applicability, water contact angle measurement, equilibrium swelling ratio analysis, and dye releasing efficiency experiment were also conducted.

**Figure 1:**
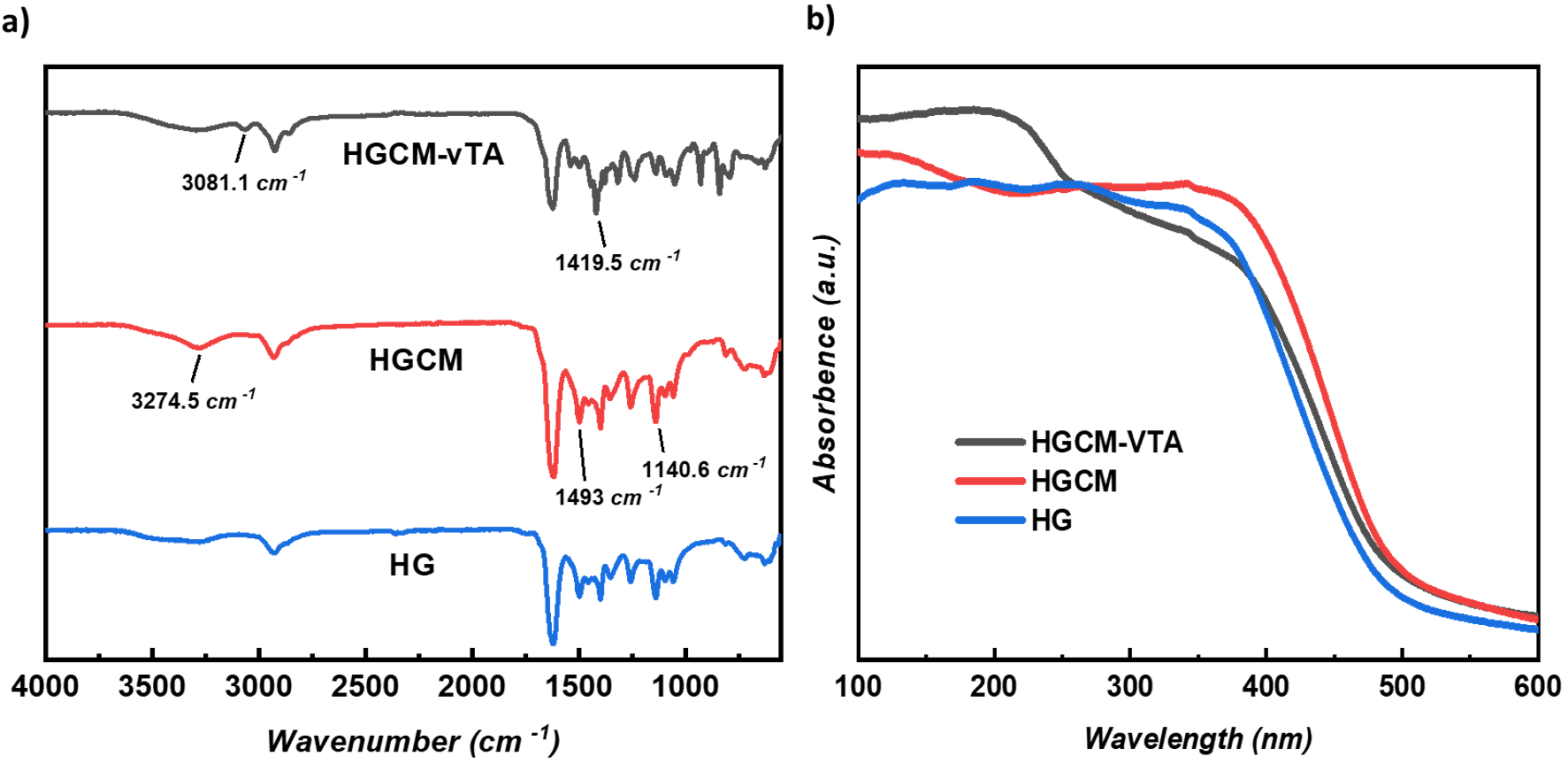
a) FTIR spectra of freeze-dried HGCM-vTA, HGCM and HG. b) UV spectra of freeze-dried HGCM-vTA, HGCM and HG.

All hydrogel samples were characterized via FTIR analysis to elucidate structural footprints of CM embedding and vTA photographing. The broad peak in the range from 3639 cm^−1^ to 3136 cm^−1^ corresponds to the hydrogen bonding between carboxyl and hydroxy groups with amide functionality of the hydrogel backbone. Significant stretching at 1620 cm^−1^ is typical for carbonyl groups of amides (Figure 1a). Thereupon, characteristic hydrogel vibrations explicitly vary according to the applied processes. The major difference between HG and HGCM is the significant peak sharpness around 3274 cm^-1^, which can be related to –NH_2_ functional group stretching relying on buried g-CN structure in hydrogel network. The vTA photografting can be revealed via distinctive signals such as the emergent peak at 3081 cm^−1^, corresponding to C–H aromatic stretchings arising from the thiazole ring, the neck at 1687 cm^−1^ signifies the C=N vibration band, and at last the intensified peak at 1419 cm^−1^ indicates a C–N stretching of the thiazole ring.

Proceeding the examination by UV–vis spectroscopy, overlaid absorption spectra of the samples revealed the photophysical differences as expected, since HG does not consist of photoactive g-CN particles in contrast to HGCM and HGCM-vTA. Moreover, the altered HGCM absorbance after surface modification provides enhanced absorption in deep UV range (Figure 1b). In addition, digital images of HG, HGCM, HGCM-vTA under UV light irradiation also reveal their emissive properties (Supporting Information File 1, Figure S1).

**Figure 2:**
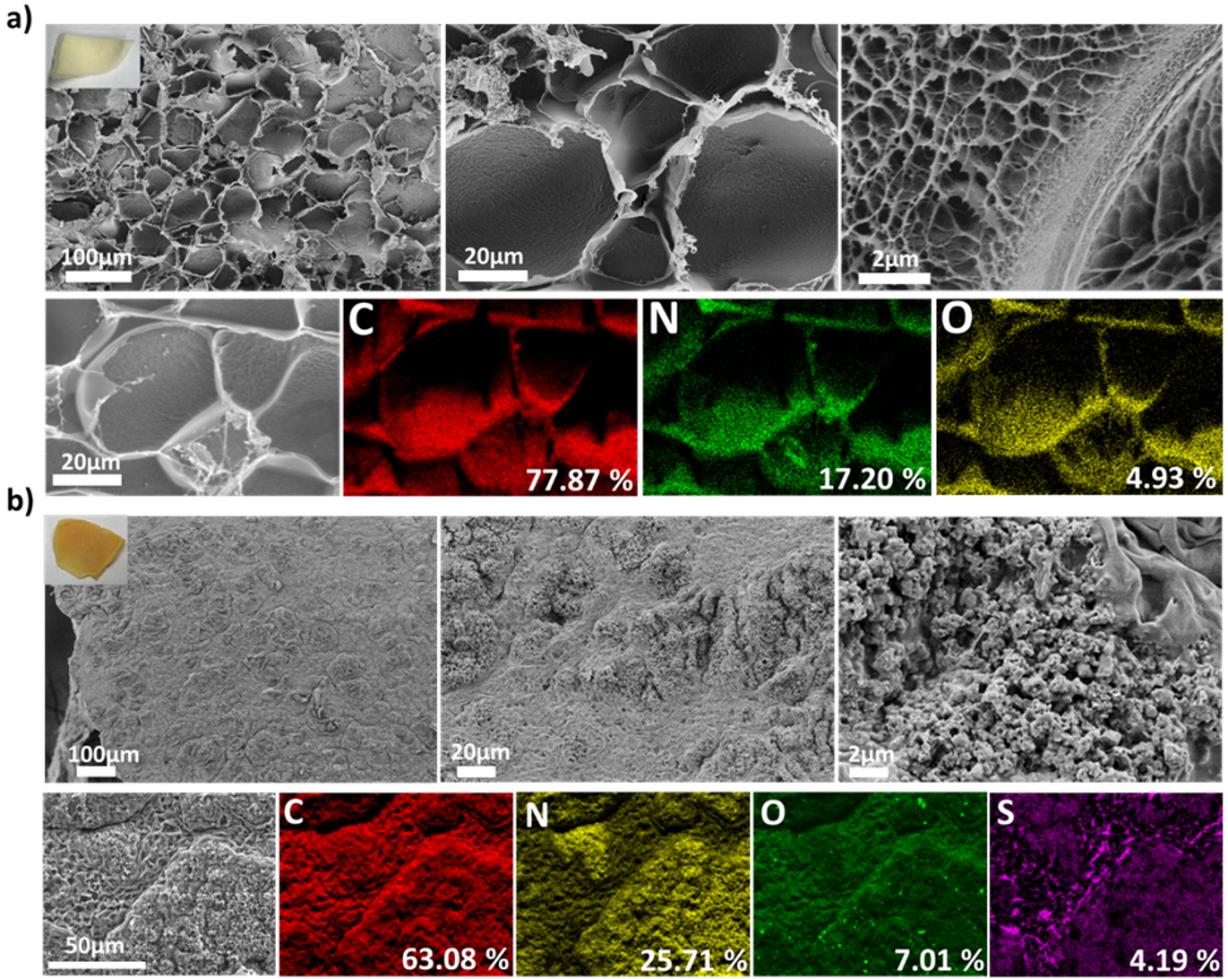
Scanning electron microscopy (SEM) images of a) HGCM and b) HGCM-vTA in combination with their elemental mapping results via EDX.

Scanning electron microscopy was performed to investigate the morphology of freeze-dried HGCM and HGCM-vTA. As shown in Figure 2a, HGCM exhibits a significantly porous and uniform morphology. Formation of accessible pores for vTA was the key point to reach in order to activate g-CN nanosheets buried in the hydrogel to perform a photoinduced modification. It must be mentioned that the light transmission is limited since the hydrogel substrate is not fully transparent, and this envisions the importance of porosity as a key factor to diffuse and reflect the incoming light that can reach embedded g-CN nanosheets to enhance the modification.

Surface transformation has drastically changed the parental hydrogel morphology. Closure of pores (Figure 2) are the supportive evidences for a successful modification as they are not observed in parental hydrogel (Figure 2a). Besides, elemental mapping results of HGCM-vTA are exhibiting a sulfur content allocation in accordance with the nitrogen atom distribution in a fair amount of abundance (Figure 2b). Determination of the sulfur content is a facile approval for the surface modification as neither monomers nor g-CN from HGCM are possessing a sulfur atom (Figure 2a).

As we attempted to modify HGCM surface from hydrophilic to hydrophobic, the corresponding material surface wettability is expected to be influenced. To investigate this, wet forms of HGCM and HGCM-vTA were subjected to a water contact angle (WCA) measurement by utilizing multiple contact points (Supporting Information File 1, Figure S2). The most stable result in terms of smooth and unwrinkled contact point of HGCM-vTA has resulted in 58.5° over 40 seconds, and the other spots have resulted in 77.8° and 68.3°. On the other hand, HGCM showed a super hydrophilic character by imbibing the water with high-speed that even imaging was not possible (see the video provided as Supporting Information File 2).

**Figure 3:**
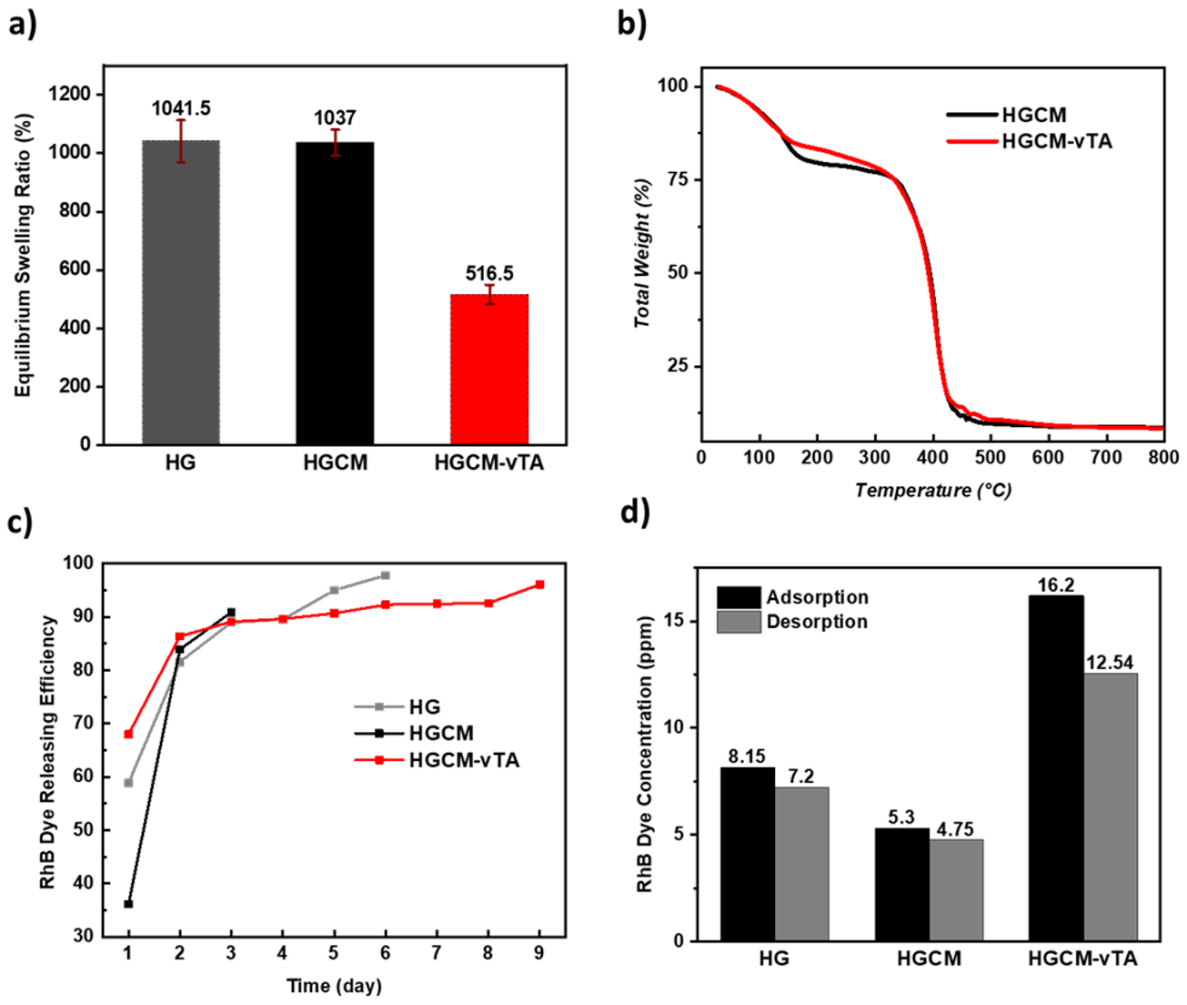
a) Equilibrium swelling ratios of HG, HGCM, HGCM-vTA at specified time intervals. b) Thermogravimetric analysis of HGCM and HGCM-vTA. c) RhB dye releasing efficiency versus time plot of HG, HGCM, and HGCM-vTA. d) RhB dye adsorption/desorption concentrations of HG, HGCM, HGCM-vTA.

It is known that the competition between the hydrophobic and hydrophilic character of hydrogels has a role in affecting water absorption and retention. In this regard, synthesized hydrogels were subjected to a swelling measurement to reveal their overall water affinity and then a TGA measurement was conducted to examine the water retention performances. The swelling ratio results showed that the surface hydrophobization led to a significant decrease, nearly a half performance, compared to unmodified samples (Figure 3a). Following that, water retention performances of HGCM and HGCM-vTA were investigated after leaving the samples to dry at room temperature for 2 days before conducting the TGA measurement (Figure 3b). TGA profiles did not indicate a significant difference for water retention, but thermal stability of HGCM-vTA within the range of 150 °C to 318 °C has improved. Considering the water retention, the first trials of the hydrophobization process were designed to perform the hydrophobization with the swollen sample instead of using freeze-dried versions to take the advantage of entrapped water that might provide the opportunity to control the water retention. Unfortunately, during the photomodification, diffusion of vTA molecules has driven the water molecules outside the network. Despite this fact, the wet-modified sample resulted in a fair water contact angle result, but, however, not enhanced water retention. Another parameter on the water swelling was the effect of g-CN presence. It was observed that the presence of g-CN in hydrogel did not affect the water swelling ratio compared to the reference hydrogel, which is reasonable in terms of the physicochemical nature of hydrophilic hydrogels.

After the swelling experiment, samples were contacted to an aqueous RhB dye solution to investigate their dye releasing efficiency by gradually washing out the samples, in order to mimic a drug or nutrient release. One of the attractive applications of g-CN relies on the adsorption and/or photodegradation of dyes. In general, these performances are reported on bulk g-CN materials, such as the on a self-standing ultra-long porous g-CN wires exhibiting outstanding RhB adsorption values by minimized solvent uptakes [46]. In our case, we focus on the RhB releasing profiles of RhB swollen hydrogels. HG, HGCM, and HGCM-vTA have been immersed in RhB solution for 24 hours, then washed with distilled water every 24 hours, and all collected samples were investigated by UV–vis spectroscopy until reaching the minimum dye absorbance (Figure 3c,d, Supporting Information File 1, Figure S4). According to released RhB dye concentration versus time plots of all samples, HGCM-vTA completed dye releasing with the longest time period of 9 days. It was followed by HG, which resulted in 6 days whereas HGCM was accomplishing the process in 3 days (Figure 3c). Besides, according to periodically collected RhB dye concentrations summed up and compared with adsorption concentrations for each sample (stock RhB concentration: 40 ppm), HG released 88% of adsorpt RhB dye in 6 days, HGCM 89% in 3 days, and HGCM-vTA 77% in 9 days (Figure 3d). In addition to RhB dye, albumin–fluorescein isothiocyanate and fluorescein isothiocyanate–dextran fluorescent probes were subjected to HGCM and HGCM-vTA samples with a comparatively shorter releasing experiment than the one performed for RhB dye (Supporting Information File 1, Figure S4a). The static experiment principally relies on physical adsorption/releasing performance being under the control of intermolecular interactions. Hydrophobization might enhance the hydrophobic interactions between the dye core and the polymer network, thus releasing can be achieved in a longer term. As a last simulative experiment, cation (K^+^, Ca^2+^, Mg^2+^) releasing performances of HGCM and HGCM-vTA after an overnight immersion in separately prepared stock solutions were analysed via ICP-OES (Supporting Information File 1, Figure S4b). HGCM shows higher cation releasing which might be driven by osmosis, however, HGCM-vTA retains cations thus offering a slower release which is highly beneficial for agricultural delivery systems.

The grand outcome of embedded g-CN-based surface photomodification has significant advantages in terms of its non-toxic process and cost-efficient material resources.

### Pore substructuring

**Scheme 3:**
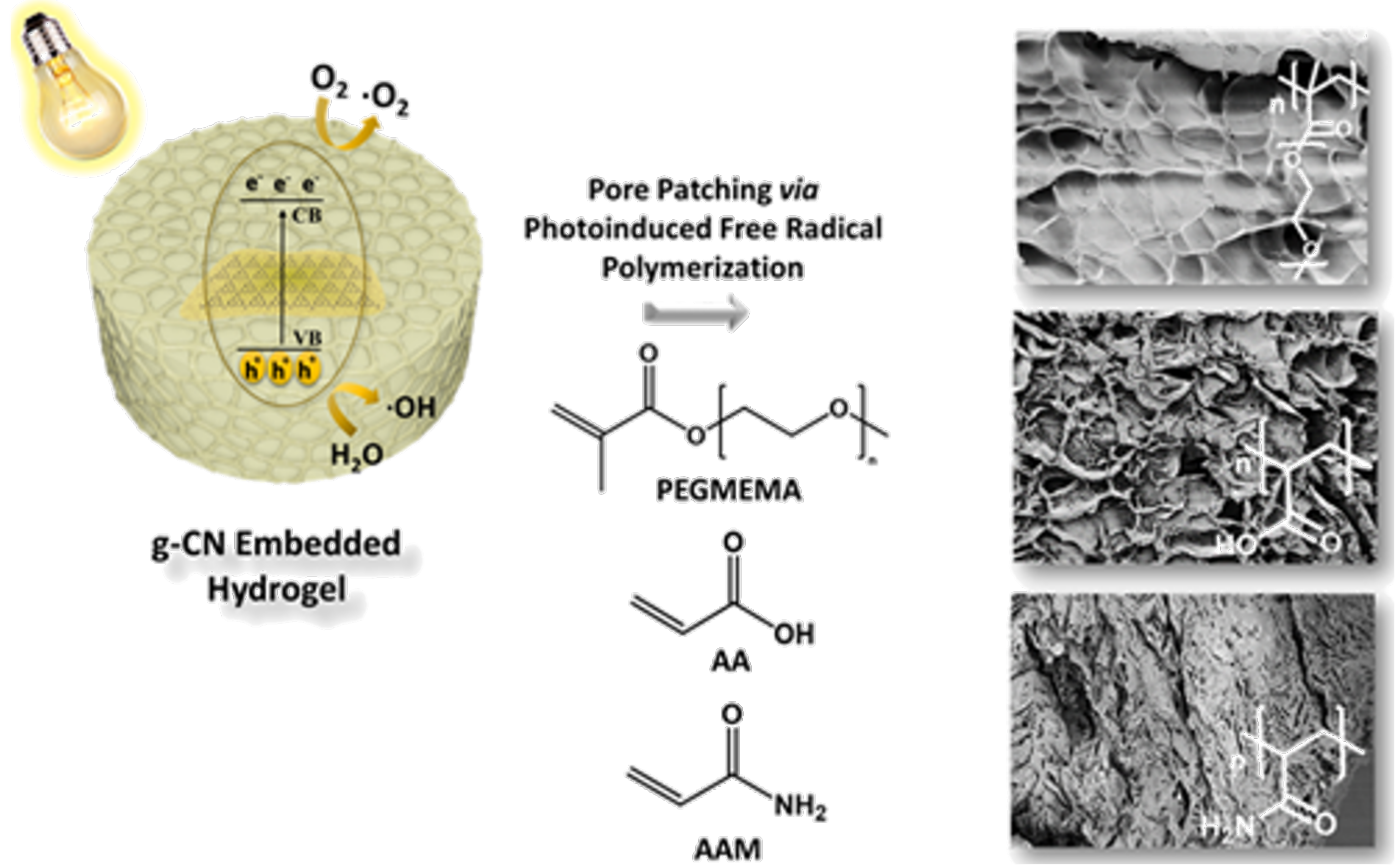
Overview of pore substructuring via photoinduced free radical polymerization over embedded g-CN nanosheets in hydrogel network.

In porous materials, the functionality of the pores is responsible for the main catalytic activity, such as in carbonaceous materials [47–48]. When a network with full functionality cannot be formed easily, one can form a rigid neutral host and modify the pores subsequently. Herein, the nanoporous system is magnified to macropores in hydrogel systems as a representative synthetic approach to modulate porous structures of hydrogels with secondary polymers via visible-light-induced reaction. The photoactivity of g-CN materials are attractive in this sense since covalent modification of the surface of g-CN has offered a versatile post modification platform [49]. The as-prepared hydrogel network (HGCM) was immersed in various acrylic monomers. Following that, photoinduced free radical polymerization of employed monomers performed under visible light irradiation by taking advantage of embedded g-CN nanosheets in HGCM. According to literature, g-CN as an organic semiconductor is utilized in polymerization processes as a photoinitiator by generation of reactive radical species (O_2_**^−•^**, HO^•^, HO_2_^•^) under convenient light illumination. The ability of g-CN to initiate polymerization and act as a polymerization locus for a covalent polymer growth have been investigated in detail in literature [37,50]. Inspired by these, we now attempt to conduct a pore modification on g-CN-embedded hydrogels by visible-light-induced reaction. HGCM was a host network and the variety of monomers were swollen in the network, polymerized, and purified (Scheme 3). After the polymerization was completed, polymer networks were purified as delineated in the experimental section. Altered pore morphology was investigated by SEM, and functional group analysis was achieved by FTIR.

**Figure 4:**
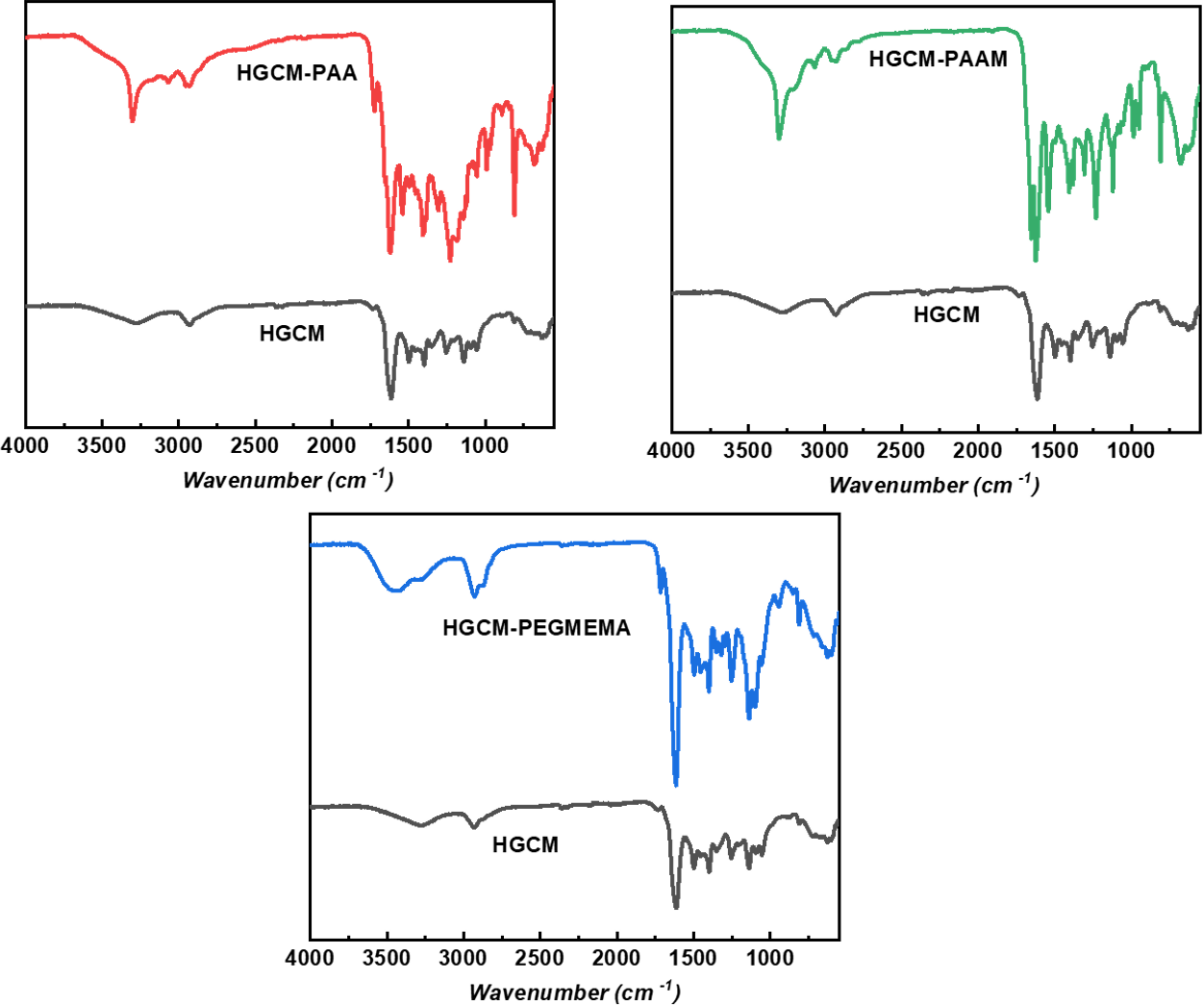
FTIR spectra of freeze-dried HGCM-PAA, HGCM-PAAM, HGCM-PEGMEMA in comparison with HGCM.

Enrichment of the hydrogel network with subsequent radical polymerization using various acrylic monomers possessing different functional groups were explicitly confirmed via FTIR (Figure 4). Regarding the polyacrylic acid-based system (PAA), the broadened signal from 2730 cm^−1^ to 3703 cm^−1^ and sharp signal at 3303 cm^−1^ corresponds to O–H stretching of the carboxylic acid group. The peak appearing at 1723 cm^−1^ can be attributed to asymmetric C=O group stretching vibrations of carboxylic acid groups on PAA chains. The polyacrylamide-based (PAAM) pore substructure spectrum is exhibiting the typical –NH stretching vibrations at 3420 cm^−1^ and the band at 1656 cm^−1^ is corresponding to the strong primary amide C=O stretching vibration of the amide group. The strong signals at 1558 and 1404 cm^−1^ are originated from amine N–H bending and scissoring –CH_2_– vibrations, respectively. At last, the poly(ethylene glycol) methyl ether methacrylate (PEGMEMA)-based network displays O–H stretching from 3106 cm^−1^ to 3710 cm^−1^ relying on pendant hydroxy groups of the PEG structure, the signal at 1719 cm^−1^ is corresponding to the C=O group stretching and the more pronounced intensities from 980 cm^−1^ to 1202 cm^−1^ are attributed to C–H bending and C–O stretching vibrations. Consequently, all FTIR spectra results confirm the photoinduced polymerization within the hydrogel network.

**Figure 5:**
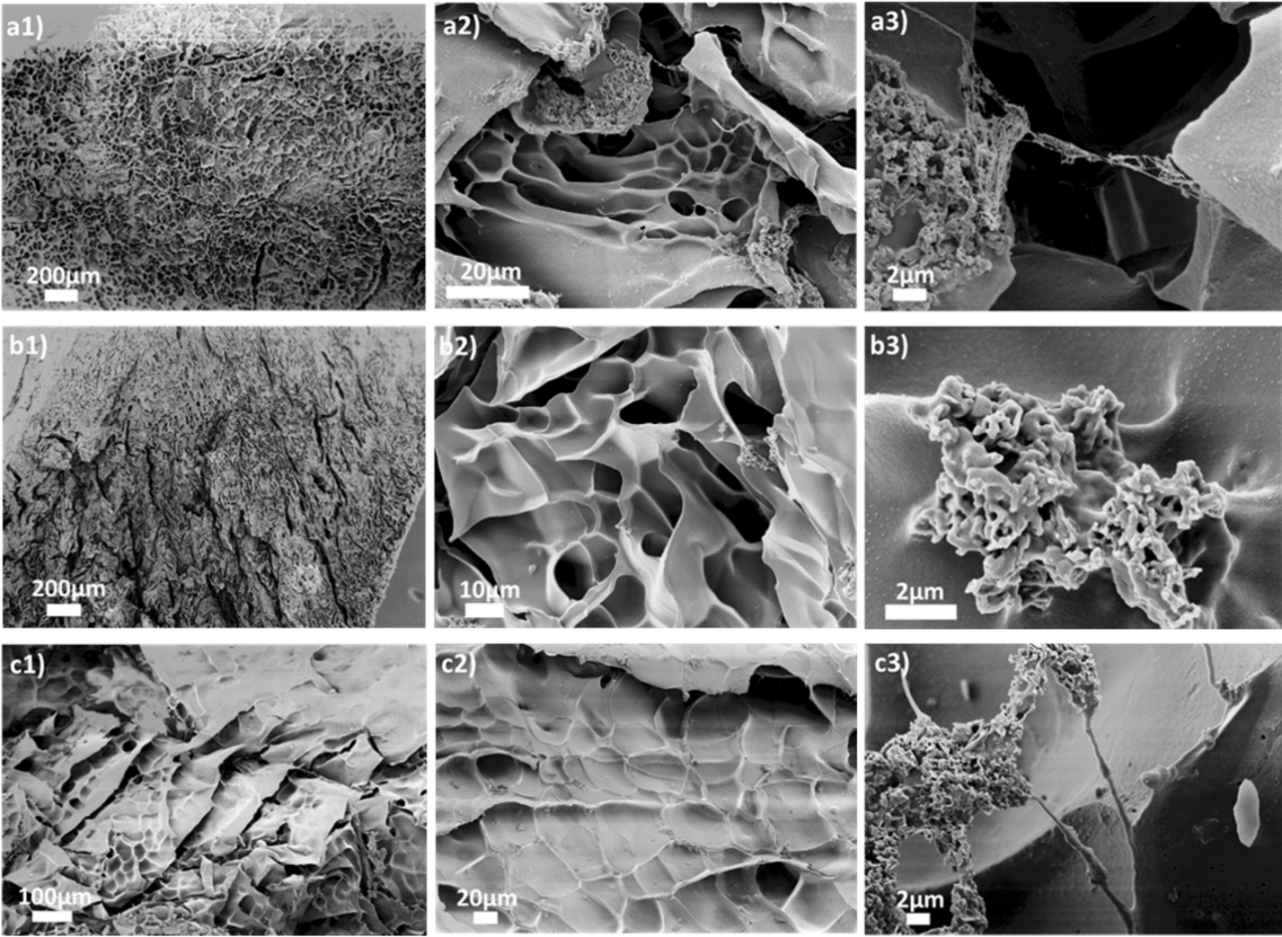
Scanning electron microscopy (SEM) images of a) HGCM-PAA, b) HGCM-PAAM, and c) HGCM-PEGMEMA.

Visible light irradiation leads to a polymerization of monomers within the three‐dimensional hydrogel network, thus one can expect altered pore morphologies. In that regard, SEM images are useful to explore the pore substructuring for each type of polymer, which are compacted and attached to the hydrogel skeleton (Figure 5). The morphology of HGCM as a main substructure was already investigated in the previous chapter. Considering the HGCM porous structure as a reference point, HGCM derived hydrogels demonstrated altered pore structures based on the type of the interpenetrated polymer. While PAA and PEGMEMA formations mostly bound intrinsically surface-attached, PAAM exhibited comb-like strands by connecting two junction points on the substrate. The reason of this might be both topological selectivity of polymers and the arbitrary photopolymerization process. In addition, we were not able to identify free polymer chains during the purification process, which indicates a covalent growth of polymers on a hydrogel host.

**Figure 6:**
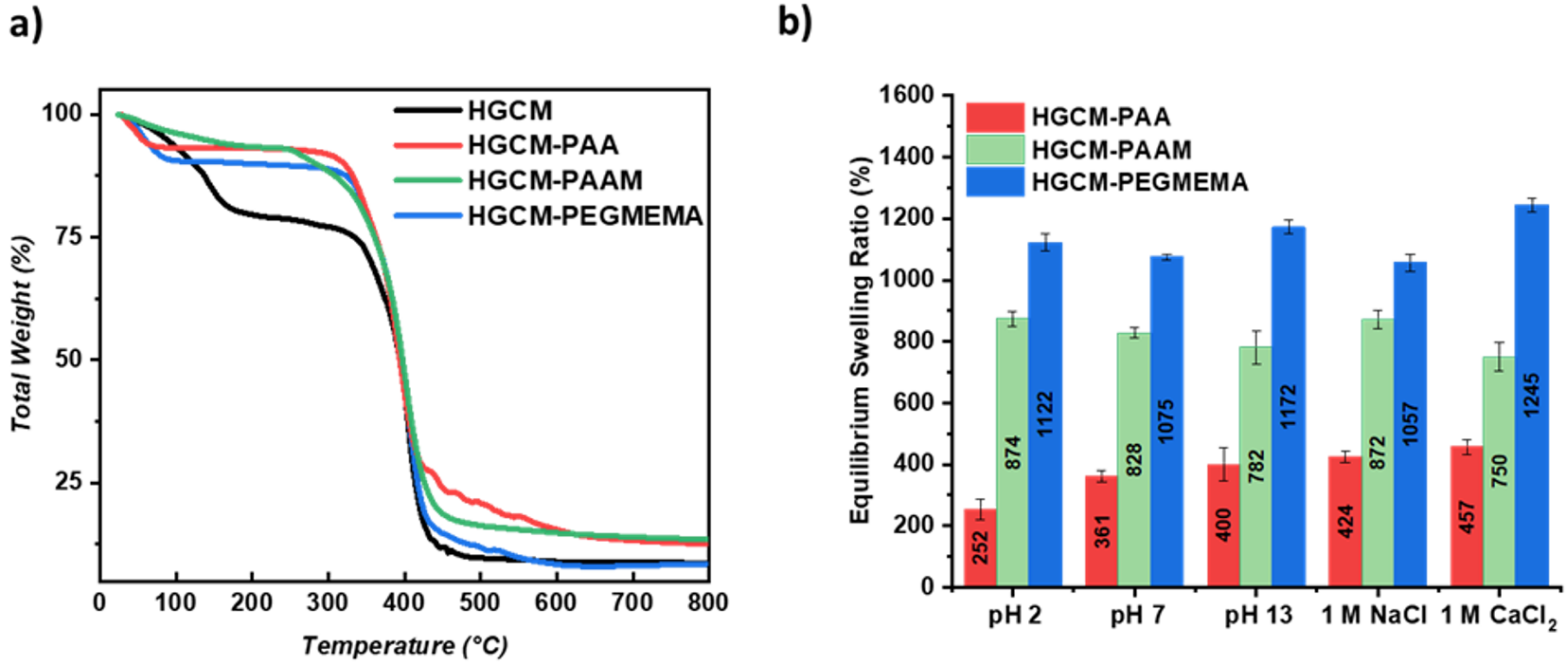
a) Thermogravimetric analysis of HGCM, HGCM-PAA, HGCM-PAAM and HGCM-PEGMEMA. b) Equilibrium swelling ratio results of HGCM-PAA, HGCM-PAAM, HGCM-PEGMEMA in various conditions; pH 2, pH 7, pH 13, 1 M NaCl, 1 M CaCl_2_.

It is expected that pore modification on networks will influence the thermal stability of the initial substrate. Regarding this, thermogravimetric analysis results confirmed the enhanced thermal stability for each employed polymer network compared to the initial substrate (Figure 6a). The HGCM curve is indicating the evaporation of entrapped water (up to 150 °C) followed by structural decomposition starting from 307 °C and ending up with 8.44% total weight at 800 °C, at first place. HGCM-PAA maintained thermal stability up to 305 °C with 8.4% mass change and resulted in 12.6% total weight whereas HGCM-PAAM was stable up to 250 °C with 7% mass change ending up with 13.4% total weight. At last, HGCM-PEGMEMA exhibited thermal stability up to 307 °C with 13% mass change and resulted in 8.5% total weight. When the temperature range between 160 °C and 300 °C is subjected for all curves, it can be concluded that the occupied pores by subsequent polymer networks are providing the thermal stability to parental HGCM substrate.

Employing a diverse set of monomers to HGCM network can have an influence on the swelling properties as well. Regarding this, swelling performances of so-formed networks were examined via swelling process at equilibrium state in various conditions (Figure 6b). The first overall interpretation is that the condition variety did not drastically affect ESR results of each sample. HGCM-PEGMEMA has the highest ESR in every condition that is in good agreement with the nonionic and highly flexible structure of PEG that can resist electrolytes and pH stimuli.

On the other hand, PAAM and PAA depicted significantly decreased ESR results, which are confirming the subsequent network formation by occupying the pores. The gap between the ESR results in each network could be considered as their patching efficiency. HGCM-PAAM performed a slight decrease from low pH to high pH value due to amine groups' protonation on the polymer backbone via electrostatic attraction, and it responded to monovalent electrolyte with a higher ESR. With the increased pH, HGCM-PAA eventuated with enhanced ESR results arising from the dissociation of carboxylic acid groups. Swelling in saltwater conditions demonstrated similar results, yet significantly lower compared to previous examples.

In addition, the error margins of equilibrium swelling behavior were affected by isotropic accretion of polymer networks over the substrate. It is important to underline that the presented polymerization process relies on a light access in the porous network, so conducting the reaction on thin substrates is favored.

## Conclusion

Embedding photoactive g-CN nanosheets in hydrogels could be an advanced trick to access photoinduced post-modification techniques. The pores of the dried hydrogel can be filled with the precursor of a secondary network, and a visible light illumination forms an extended network with interlocked character. Alternatively, hydrophobization of a hydrogel can be attained by visible-light-induced photografting of vinylthiazole groups. In the present paper, two straightforward synthetic pathways for hydrogel post-modification are presented, and final products could be of great interest from the materials science perspective. Visible-light-induced post modification technology by semiconductors on porous networks could be extended to other porous systems as well, such as carbonecous networks.

## Experimental

**Materials:** 4-Methyl-5-vinylthiazole (vTA, 97%, Sigma-Aldrich), ʟ-(+)-ascorbic acid (AsA, 98+%, Alfa Aesar), *N*,*N*-dimethylacrylamide (99.0+%, TCI), *N*,*N′*-methylenebis(acrylamide) (MBA, 99%, Sigma-Aldrich ), acrylic acid (AA, 99%, Sigma-Aldrich), acrylamide (AAM, 98.5%, Acros), albumin–fluorescein isothiocyanate conjugate (FITC-Albumin, Sigma-Aldrich), calcium chloride (CaCl_2_, 97%, Alfa Aesar), cyanuric acid (98%, Sigma-Aldrich), fluoresceinisothiocyanat-dextran (FITC–Dextran, 10.000 *M*_w_), hydrochloric acid (HCl, 37%, Sigma-Aldrich), hydrochloric acid (1 M solution, Sigma-Aldrich), hydrogen peroxide (30% aqueous solution, Merck), magnesium chloride (MgCl_2_, 99%, Merck), melamine (99%, Sigma-Aldrich), poly(ethylene glycol) dimethacrylate (PEGDMA, *M*_n_ 550, Sigma-Aldrich), poly(ethylene glycol) methyl ether methacrylate (PEGMEMA, *M*_n_ 300, Sigma-Aldrich), potassium chloride (KCl, 99%, Merck), rhodamine B (RhB, 95%, Sigma-Aldrich), sodium chloride (NaCl, 99%, Sigma-Aldrich), sodium hydroxide (1 M solution, Sigma-Aldrich). DMA, PEGDMA, PEGMEMA and were passed through basic alumina column prior to use.

**Synthesis of g-CN:** g-CN was synthesized from cyanuric acid–melamine supramolecular complex as reported in literature [42]. Cyanuric acid (1.29 g) and melamine (1.26 g, 1:1 molar ratio) were mixed in 50 mL distilled water overnight to form cyanuric acid–melamine complex, then the solid was filtered and dried in vacuum overnight. The dried product was transferred into a capped crucible and put into an N_2_-protected oven at 550 °C for 4 hours, with a heating rate of 2.3 °C /min. The resulting yellow powder is labeled as g-CN (CM).

**Synthesis of g-CN nanosheets embedded hydrogel (HGCM):** 150 mg as-prepared CM was dispersed in 30 mL distilled water and sonicated 3 times for 30 minutes to exfoliate g-CN nanosheets (CM-W). 9 g freshly prepared CM-W, 0.8 g DMA, 0.150 g MBA and 0.150 g AsA were weighted into a flask, mixed for 5 minutes, then sonicated for 20 seconds. Following that, 1.5 mL hydrogen peroxide solution was injected into the mixture and placed in a Petri dish after mixing thoroughly. The Petri dish was capped and left for 3 hours to obtain a gelation via redox-induced free radical polymerization. Afterwards, it was washed with distilled water to remove the unreacted species and freeze dried for 24 hours. The resulting light and brittle g-CN nanosheets embedded hydrogel was ready for further usage. In addition, a comparative sample was prepared with the same procedure in the absence of CM, then the final hydrogel is denoted as (HG).

**Synthesis of hydrophobic hydrogels (HGCM-vTA):** 100 mg as-prepared HGCM and 5 mL vTA were put in a glass vial and left for 30 minutes to complete adsorption–desorption equilibrium. Afterwards, it was placed between two visible light sources (10 cm distance each) for 5 hours to perform in situ photomodification based on the photoactivity of embedded g-CN nanosheets within the hydrogel network. After 5 hours, hydrophobized HD-CM was placed in a Petri dish and washed with 20 mL acetone to remove the remaining vTA, then left in a fume hood for drying overnight.

**Pore substructuring of HGCM by photoinitiation:** 100 mg as-prepared HGCM and certain amounts of patching monomer (consisting 10 mol % crosslinker) were added into a capped glass vial containing 2 g distilled water and left for 2 hours to complete adsorption–desorption equilibrium. After 2 hours, monomer swollen hydrogel was replaced into another capped glass vial and set between two visible light sources (10 cm distance each) overnight to accomplish photopolymerization. Afterwards, it was purified by immersing in 20 mL distilled water refreshed repeatedly every 2 hours for 3 times and then left in a fume hood for drying overnight. This procedure was repeated for each substructuring monomer categorized as acidic AA (10 mol % MBA), cationic AAM (10 mol % MBA), and neutral PEGMEMA (10 mol % PEGDMA).

**Rhodamine B dye releasing experiment:** 40 mg HD, HGCM and HGCM-vTA were weighted separately in a capped glass vial containing 4 mL RhB dye solution (4 × 10^−2^ M) and left for 24 hours. Afterwards, all samples were replaced in another glass vial containing 4 mL distilled water to follow the dye releasing progress spectroscopically every 24 h. The RhB dye releasing efficiency was calculated by using the following formula for each sample:


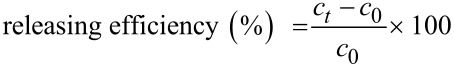


*c*_0_: initial RhB dye concentration, *c_t_*: RhB dye concentration at specified time.

**FTIC-albumin and FTIC-dextran releasing experiment:** 40 mg HGCM and HGCM-vTA were weighted separately in a capped glass vial containing 4 mL FTIC-albumin (2000 ppm) and FTIC-dextran (2000 ppm) solutions and left overnight. Afterwards, all samples were placed in another glass vial containing 4 mL distilled water to spectroscopically monitor the labeled molecule release process after 24 h.

**Cation releasing experiment:** 100 mg HGCM and HGCM-vTA were separately immersed in freshly prepared KCl, CaCl_2_ and MgCl_2_ stock solutions (corresponding concentration for each cation, K^+^, Ca^2+^ and Mg^2+^ = 1000 ppm) overnight. Afterwards, samples were placed in a capped glass vial containing 10 mL distilled water, respectively, and left overnight. Released contents of each cation were analyzed via ICP-OES.

**Characterization:** Fourier transform infrared (FTIR) spectra were acquired on a Nicolet iS 5 FT-IR spectrometer. Solid-state ultraviolet−visible (UV−vis) spectroscopy for grinded samples was performed via a Cary 500 Scan spectrophotometer equipped with an integrating sphere. Thermogravimetric analysis (TGA) was performed via TG 209 Libra from Netzsch under nitrogen atmosphere with a heating rate 10 K min^−1^ using aluminum crucible for samples. Trace analysis of potassium, calcium and magnesium cations were performed via inductively coupled plasma optical emission spectroscopy (ICP-OES Optima 8000). Scanning electron microscopy (SEM) and EDX elemental mapping were performed using a JSM-7500F (JEOL) microscope equipped with an Oxford Instruments X-Max 80 mm^2^ detector for the determination of both elemental composition and morphology. The CM/water suspension was prepared in a sonication bath at 50% amplitude from Elma (Transsonic T310). Freeze drying was applied to hydrogels for 24 hours (LSC, Christ, Germany) in order to obtain solid samples with protected porous architectures for further investigations. Water contact angle measurement was performed using a Krüss contact angle measuring system G10 and recorded via Krüss official software. The sample with ideally flat surface is placed in front of a camera which records the water drop on the surface and estimates the angle between water droplet and surface. This method is useful to determine surface properties, such as hydrophilicity and hydrophobicity in the present case.

Swelling ratio analysis of the samples were calculated with the following procedure: 40 mg dried hydrogels (*Wd*) were put into separate vials containing 4 mL of deionized water at pH 2, pH 7, and pH 13 (prepared from 1 M HCL and 1 M NaOH stock solutions), 1 M NaCl solution and 1 M CaCl_2_ solution, respectively. The vials are then capped and left for different time intervals taking into consideration of equilibrium swelling point at room temperature. Relative standard deviation was calculated based on the measurement of 3 samples. Hydrogels treated with solutions were weighted separately (*Ws*), and the swelling ratio was calculated by using the following formula for each sample:


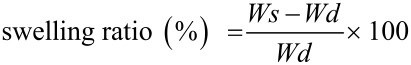


## Supporting Information

File 1 Four supporting figures.

File 2 Water contact angle video.
