# Supplementary material for: Photoinduced post-modification of graphitic carbon nitride-embedded hydrogels: synthesis of 'hydrophobic hydrogels' and pore substructuring
Source: Beilstein J Org Chem. 2021 May 21;17:1323–34. doi: 10.3762/bjoc.17.92 (PMC8182687; doi:10.3762/bjoc.17.92)
Supplement: File 1 — Four supporting figures. [file Beilstein_J_Org_Chem-17-1323-s001.pdf]

BEILSTEIN JOURNAL OF ORGANIC CHEMISTRY

# Supporting Information

for

# Photoinduced post-modification of graphitic carbon nitride-embedded hydrogels: synthesis of 'hydrophobic hydrogels' and pore substructuring

Cansu Esen and Baris Kumru

*Beilstein J. Org. Chem.* **2021,** *17,* 1323–1334. doi:10.3762/bjoc.17.92

## Four supporting figures

License and Terms: This is a supporting information file under the terms of the Creative Commons Attribution License (https://creativecommons.org/licenses/by/4.0). Please note that the reuse, redistribution and reproduction in particular requires that the author(s) and source are credited and that individual graphics may be subject to special legal provisions.
The license is subject to the *Beilstein Journal of Organic Chemistry* terms and conditions: (https://www.beilstein-journals.org/bjoc/terms)

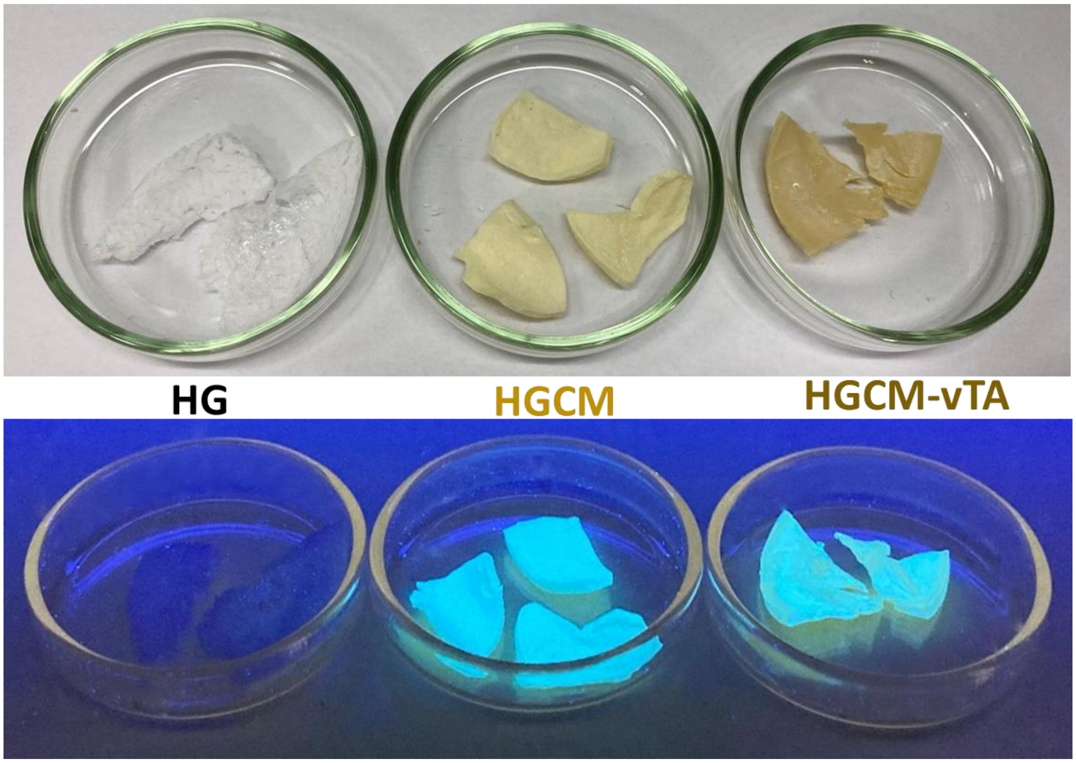


**Figure S1:** Digital images of HG, HGCM and HGCM-vTA under daylight and UV light illumination.

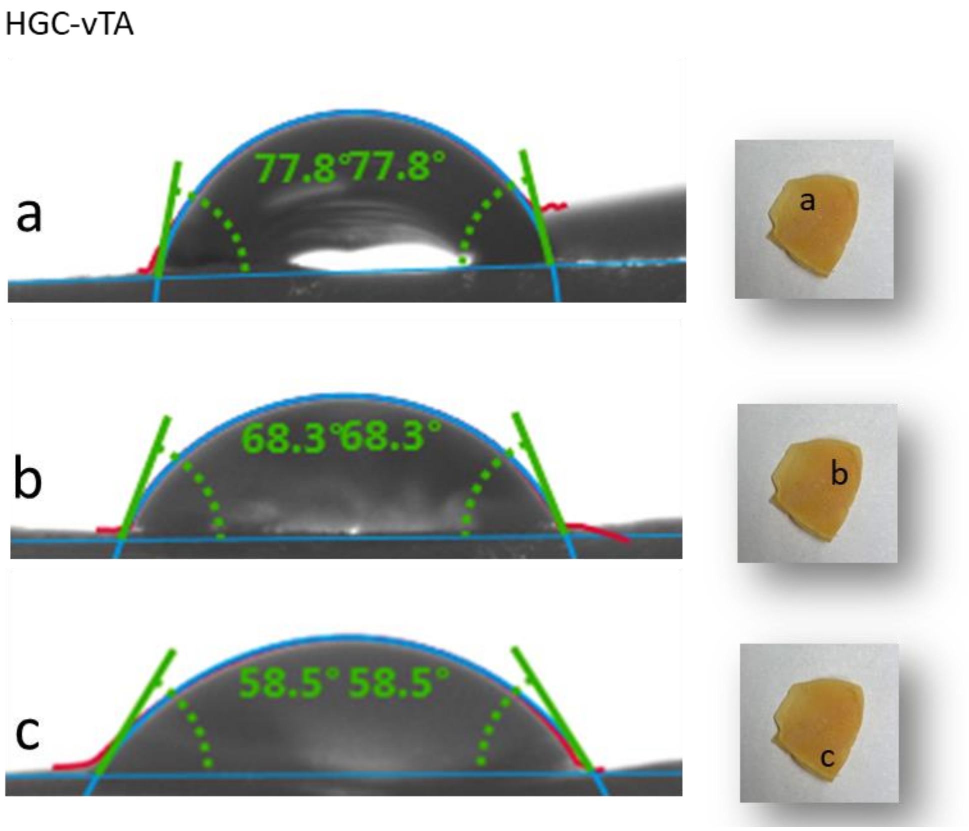


**Figure S2:** Water contact angle results of HGCM-vTA on multiple contact points (obtained after 15 seconds of equilibrium).

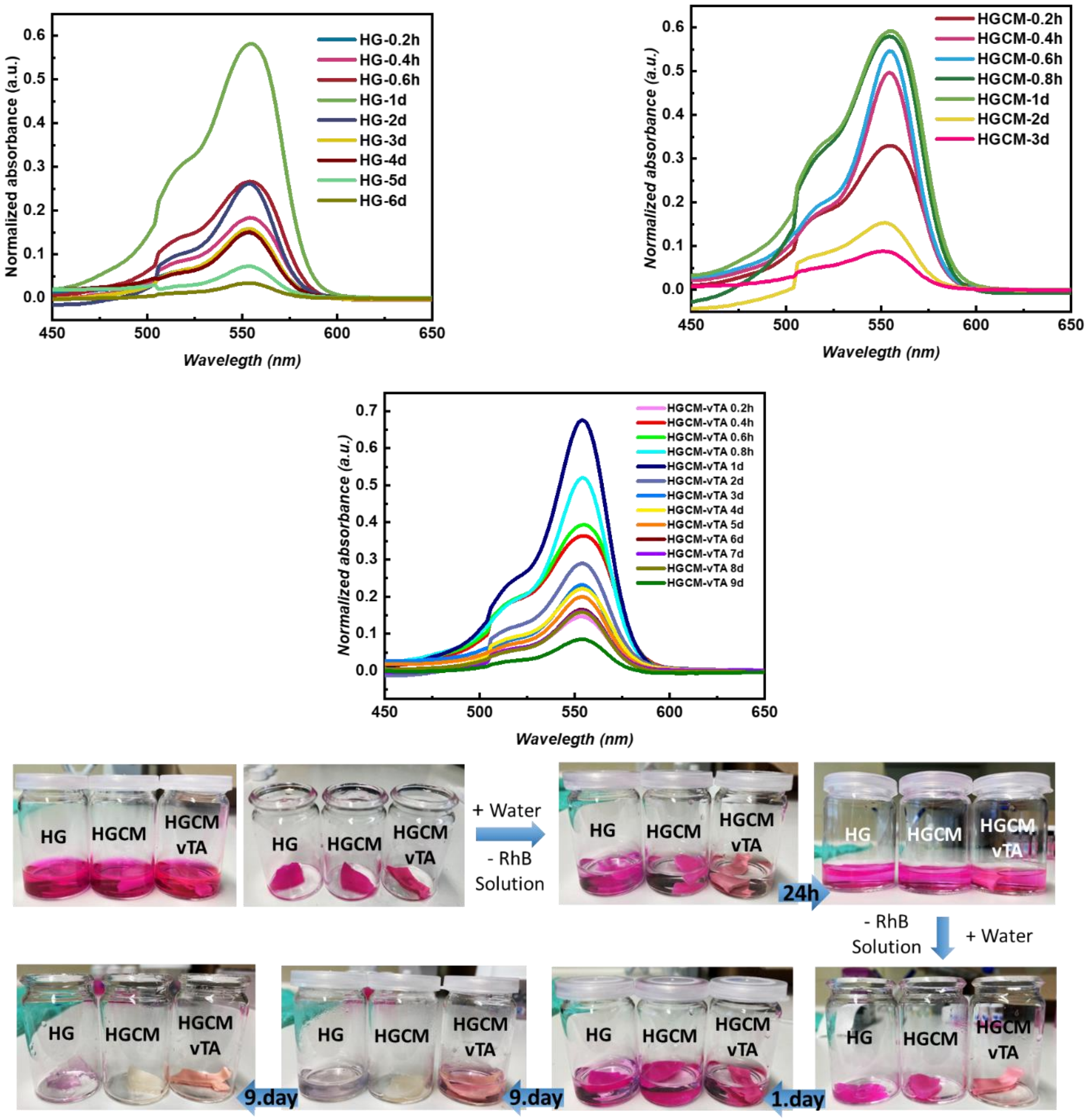


**Figure S3:** RhB dye releasing progress of HG, HGCM and HGCM-vTA at specified time intervals followed by UV–vis spectroscopy.

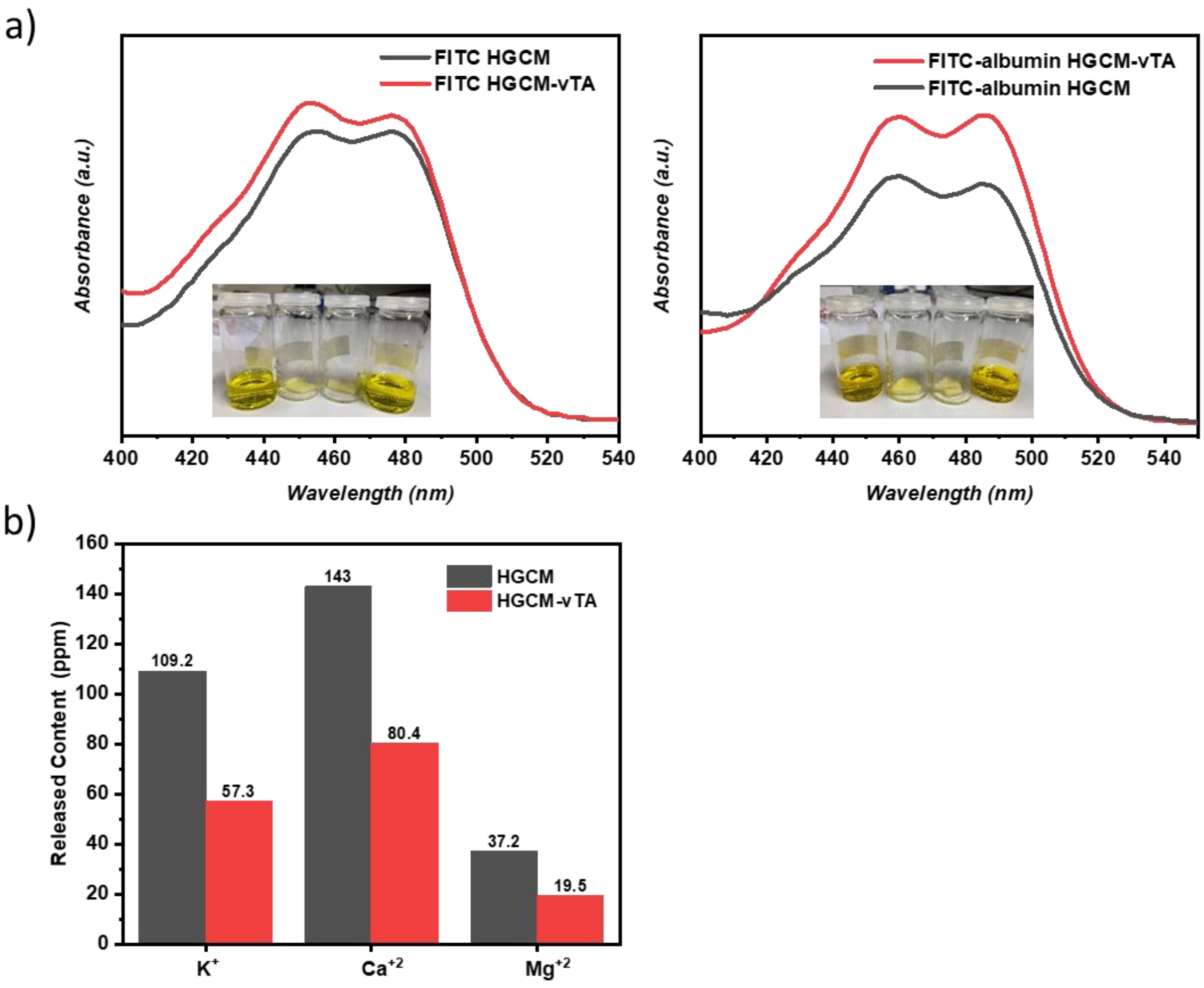


**Figure S4:** a) FITC-dextran and FITC-albumin releasing progress (after 24 hours) of HGCM and HGCM-vTA monitored by UV–vis spectroscopy. b) Inductively coupled plasma optical emission spectroscopy (ICP-OES) results of released $K^+$, $Ca^{2+}$ and $Mg^{2+}$ content for HGCM and HGCM-vTA.
